# Surgical complications in 550 consecutive cochlear implantation

**DOI:** 10.1590/S1808-86942012000300014

**Published:** 2015-10-14

**Authors:** Rubens Brito, Tatiana Alves Monteiro, Aquiles Figueiredo Leal, Robinson Koji Tsuji, Mariana Hausen Pinna, Ricardo Ferreira Bento

**Affiliations:** a Senior Associate Professor of Otorhinolaryngology – Medical School of the University of São Paulo (HCFMUSP).; b MD. Fellow in Ear Surgery and Lateral Skull Base.; c PhD student; Assistant ENT physician at the Craniofacial Anomalies Rehabilitation Hospital.; d PhD in Sciences (Assistant Physician – Department of Otorhinolaryngology - HCFMUSP).; e PhD student (Assistant Physician – Department of Otorhinolaryngology - HCFMUSP).; f Senior Associate Professor of Otorhinolaryngology – HCFMUSP. University Hospital – Medical School of the University of São Paulo.

**Keywords:** cochlear implants, deafness, intraoperative complications, postoperative complications

## Abstract

Cochlear implantation is a safe and reliable method for auditory restoration in patients with severe to profound hearing loss.

**Objective:**

To describe the surgical complications of cochlear implantation.

**Materials and Methods:**

Information from 591 consecutive multichannel cochlear implant surgeries were retrospectively analyzed. All patients were followed-up for at least one year. Forty-one patients were excluded because of missing data, follow-up loss or middle fossa approach.

**Results:**

Of 550 cochlear implantation analyzed, 341 were performed in children or adolescents, and 209 in adults. The mean hearing loss time was 6.3 ± 6.7 years for prelingual loss and 12.1 ± 11.6 years for postlingual. Mean follow-up was 3.9 ± 2.8 years. Major complications occurred in 8.9% and minor in 7.8%. Problems during electrode insertion (3.8%) were the most frequent major complication followed by flap dehiscence (1.4%). Temporary facial palsy (2.2%), canal-wall lesion (2.2%) and tympanic membrane lesion (1.8%) were the more frequent minor complications. No death occurred.

**Conclusion:**

There was a low rate of surgical complications, most of them been successfully managed. These results confirm that cochlear implant is a safe surgery and most surgical complications can be managed with conservative measures or minimal intervention.

## INTRODUCTION

Cochlear Implant (CI) surgery is an effective and doable alternative to restore hearing in cases of bilateral severe to profound hearing loss in patients who do not benefit from using an individual sound amplification device (ISAD)^1^. It is a complex procedure, one that combines conventional ear surgery techniques with exclusive maneuvers for this type of procedure^2^, ^3^.

The first CI surgery in Brazil was carried out in the late 70's^4^. However, its regulation and the implementation of the Brazilian Cochlear Implant Program started only in 1999, with the publication of ordinance number 1,278 by the Department of Health^5^.

It is estimated that there are over 150,000 CI surgeries made in the world and, in Brazil, this number is estimated to be 2,000^6^. Cohen et al.^7^ were the first to describe the complications stemming from the Multichannel Cochlear Implant Surgery in a representative sample. According to the literature, the global rate of complications varies between 4.7% and 40%, and the description of these complications is very heterogeneous, without an established pattern^8^, ^9^, ^10^, ^11^, ^12^, ^13^, ^14^, ^15^. So far, we do not have national data on cochlear implant surgery complications in the long run.

Therefore, the goal of the present study was to analyze the prevalence of complications associated with cochlear implant surgeries carried out in a tertiary reference center with an ear surgery training program. Moreover, we characterized the most prevalent complications and the final outcome implications associated with cochlear implant.

## MATERIALS AND METHODS

### Series

We surveyed data concerning 591 consecutive multichannel cochlear implant surgeries, between April of 1999 and May of 2010, carried out in the Cochlear Implant Group of the Teaching Hospital of the University of São Paulo Medical School - HCFMUSP. The cochlear implant database of the HCFMUSP was created in 2005 and the information was retrospectively inserted in the period before its creation, by means of reviewing the old charts and forms. As of 2005, the data was prospectively inserted in routine fashion.

All the patient candidates to cochlear implants signed the informed consent form before the surgery. The follow-up time was of at least one year of post-op.

We excluded 41 patients because of incomplete data, loss of follow up, access through the middle fossa, first surgery in another service or another native language.

### Surgical procedure

The approach was transmastoid with posterior myringotomy. Surgery was bilateral in 29 cases, 15 simultaneously and 14 were sequentially done. The Inverted “S” or small inverted “S” was done until the year of 2005. After that we started doing the minimum incision, especially in smaller children, the so-called “C-shaped” incision.

The patients were routinely given intravenous ceftriaxone or cephalexin per os during one week in the post-op. X-rays in the transorbital and Stenvers views were done in all the patients before discharge, in order to check the position of the electrode bundle. A specialist speech and hearing therapist checked the electrode impedance values and intraoperative neural telemetry.

Surgical complications were classified according to the criteria from Cohen & Hoffman^12^, which divided them into: major, when there is the need for surgical intervention or hospital admission for problem solution; and lesser, when outpatient treatment and observation only are enough. Besides immediate complications, long-term complications are also described.

This study was approved by the Ethics Committee for the Assessment of Research Projects of the Medical School of the University of São Paulo, under protocol number 0075/11.

### Statistical analysis

The analyzed data included information on: age; gender; hearing loss type and etiology; follow up time; intraoperative analysis and postoperative complications. The variables were analyzed in a descriptive fashion; for the quantitative variables we calculated the mean, standard deviation and median; and for the qualitative ones, the absolute and relative frequencies.

## RESULTS

### Clinical-epidemiological characteristics

The patients seen in the HCFMUSP Implant Group came from all over Brazil, except Roraima and Acre. The South and Southeastern regions corresponded to 84% (461) of the cases, followed by the Northeast with 8% (44), North with 3% (19) and Mid-west with 5% (26).

Of the 550 surgeries analyzed, the ratio of men and women was very similar (47.5% and 52.5%, respectively). The pediatric range of patients (up to 18 years of age), corresponded to more than half of the operated cases (331), followed by adults (197) and only 22 elderly (older than 60 years) (Table 1). The mean time of follow up was 3.9 ± 2.8 (1-12) years.

**Table 1:** Major complications.

Number of complications (%)
Electrode insertion problems 21 (3.8%)
Electrode – wrong position 11
Damaged electrode 1
Compressed electrode 2
Insertion failure 6
Shifting 1
Flap dehiscence/infection 8 (1.4%)
Cholesteatoma 6 (1.1%)
Otomastoiditis 6 (1.1%)
Peripheral facial paralysis with sequela 5 (0.9%)
CSF fistula 2 (0.4%)
Meningitis 1 (0.2%)
Disabling otological symptoms 1 (0.2%)

Total 49 (8.9%)

Pre/perilingual hearing loss corresponded to 61.6% (339) and postlingual to 38.4% (211) of the cases. The mean hearing loss time in pre/perilingual patients was of 6.3 ± 6.7 (1-48) years and of 12.1 ± 11.6 (0.8-54) years in the postlingual. The idiopathic etiology was the most frequent: 40.5% (223) followed by meningitis 18.4% (101), infectious viral causes 7.3% (40), ototoxicity 6.4% (35), genetic 6.0% (33), traumatic 4.5% (25), otosclerosis 4.0% (22) and malformations 3.3% (18) (Table 1).

The devices utilized were: Nucleus 22 - Cochlear Corporation, Australia (n = 50), Nucleus 24K/M - Cochlear Corporation, Australia (n = 160), Nucleus 24 Double Array - Cochlear Corporation, Australia (n = 12), Nucleus 24 Contour - Cochlear Corporation, Australia (n = 84), Nucleus Freedom - Cochlear Corporation, Australia (n = 149), Hires 90K - Advanced Bionics, USA (n = 17) e Medel Sonata - Medel Electronics, Austria (n = 72). In six patients it was not possible to insert the bundle of electrodes because of the lack of cochlear patency.

### Surgical complications

Among the 550 cochlear implant surgeries carried out, there were 92 complications. Major complications happened to 8.9% (49) of the procedures, a rate that was very similar to that of the minor complications: 7.8% (43). No patient died because of the cochlear implant, and only one had a life-threatening situation (meningitis), which was clinically solved.

### Major complications

Problems during electrode bundle insertion were the most frequent complication, happening in 21 (3.8%) cases. Of these, one bundle of electrodes was damaged during insertion, two suffered compression, 11 were wrongfully positioned and one bundle of electrodes shifted. In six cases it was not possible to insert the bundle of electrodes because of total cochlear obliteration. Flap complications were the second most frequent, infection with consequent surgical wound dehiscence happened to 1.4% (8) of the cases.

There were five cases of peripheral facial paralysis with sequela, all immediate and within the first five days of the multichannel cochlear implant program onset. Of these, four patients remained with facial paralysis according to grade III House-Brackmann scale and only one with grade V in the House-Brackmann scale.

Otomastoiditis affected six (1.1%) cases, of which five were children. In these five children, the cause was acute otitis media, resolved with hospital stay, myringotomy and intravenous antibiotic treatment with third generation cephalosporin. In adult patients, there was suppurative acute otitis media with residual perforation of the tympanic membrane. For this reason, there was a tympanoplasty, but after one year of surgery, the patient evolved with cholesteatomatous chronic otitis media. Cholesteatoma appeared later in six (1.1%) patients, with a mean time of 45 ± 31 (18-84) months after surgery. All required surgical approach for infection resolution.

The postoperative CSF fistula happened to two patients, one with post-meningitis hearing loss and one because of a CMV congenital infection and after resolution of the infection, the implant was removed. In the other one, a gusher was corrected with fat in the intraoperative, but after 15 days the patient developed a CSF fistula, being surgically treated and it was decided to obliterate the external auditory canal.

One patient with cochlear otosclerosis had disabling dizziness and tinnitus in the postoperative and poor auditory responses. Since the bundle of electrodes was mal-positioned upon the CT scan, we chose to remove the entire device.

### Minor complications

The minor complications are summarized on Table 2.

**Table 2:** Minor Complications.

Number of complications (%)
Transient Peripheral Facial Palsy 12 (2.2%)
Meatus posterior wall injury (2.2%)
Tympanic membrane/angle injury 10 (1.8%)
Perilymphatic fistula 7 (1.3%)
Hemorrhage 6 (1.1%)
Chorda tympani nerve injury 3 (0.5%)
Hematoma 3 (0.5%)

Total 43 (7.8%)

Among the minor complications, transient peripheral facial paralysis was the most frequent, happening to 2.2% (12) of the patients. It was immediate in nine patients and of late onset in four. All of them had total recovery.

Damage to the tympanic membrane or annulus happened in 1.8% (10) of the cases, all were intraoperatively corrected. Notwithstanding, three patients developed a residual perforation and were submitted to tympanoplasty, which was successful.

Injury to the posterior wall of the external acoustic meatus during surgery happened in 2.2% of the patients (12), which we corrected with bone wax or chips from cortical bone in most cases. The perilymphatic fistula which happened to seven patients was successfully corrected during surgery by means of fat tissue obliteration.

During surgery, there was hemorrhage in six patients and chorda tympani nerve injury in three. Three patients had a hematoma.

### Explant

22 patients in our sample had their implants removed, corresponding to 4.0%. Among the causes for explant, the erroneous insertion of the electrode bundle (n = 10) was the most common, followed by infection with wound dehiscence (n = 5). Explant because of a late development of a cholesteatoma happened in four cases. Explant because of a broken electrode bundle during insertion, by failure in the device and CSF fistula with consequent meningitis happened in one case each.

### Reimplant

Of the 22 explant cases, 16 were reimplanted. Of these, six were ipsilateral and 10 were contralateral.

## DISCUSSION

This is the first Brazilian study to describe the complications stemming from cochlear implant surgery in the long term. We found a complication rate higher than 8.9% and lower than 7.8% in our patients. All the major complications were clinically or surgically treated with much success, and there was no fatal outcome. Thus, the benefits of cochlear implants to hearing impaired patients justify the risks.

Although the prevalence of complications in our sample is higher than those from most studies in the literature, we believe this can be explained by the fact that we operate cases of higher complexity, with cochlear ossification and inner ear malformation^8^, ^9^, ^10^, ^11^, ^12^, ^13^, ^14^, ^15^, ^16^. Moreover, our clinic is a center for the training of ear surgeons, with a complementary specialization program, in which trainee surgeons participate actively in the cochlear implant surgeries. And, finally, because of adding the late complications, such as cholesteatoma and otomastoiditis, which make up 25% of the major complications.

A greater prevalence of major complications was seen in cases of hearing impairment caused by inner ear malformation (16.6%) and meningitis (14.8%), when compared to the general sample (8.9%). The literature discusses an increased risk of CSF fistula and facial nerve injury in those cases with inner ear malformation^16^. The perilymphatic fistula, when present, does not prevent the surgical procedure, since its intraoperative correction by means of obliteration with fascia temporalis, perichondrium or muscle is efficient in most cases^16^, ^17^. In our sample, the incidence of perilymphatic fistula was 1.1%, very similar to the rate described by Wooten et al.^17^. As it happened in their sample, all our cases were solved with the fistula obliteration during surgery, and none of the patients had meningitis.

Meningitis was the second most frequent cause of hearing loss, with a higher rate of major complications (14.8%), corresponding to 30% of the major complications in our sample. Problems during electrode bundle insertion were the most frequent complication because of cochlear ossification. Partial or complete ossification of the cochlear basal turn can be radiologically identified in 80% of the patients with hearing loss caused by meningitis^18^. In our group, the cochlear implant surgeries in ossified cochleas are carried out by experienced surgeons and, despite this, the risk of failure or complications is high. Therefore, we suggest it should not be made by beginners.

Problems during the insertion of the electrode bundle were the most frequent major complication in our sample, corresponding to 3.8% of the cases. We noticed that etiologies with a potential risk of ossifying labyrinthitis were the main causes: meningitis (12), otosclerosis (1) and chronic otitis media (1). The other cases with problems with electrode insertions happened in the Mondini malformations (1) or unknown etiology (6). Therefore, patients with hearing loss matching etiology with a potential risk for ossifying labyrinthitis must be properly counseled in the preoperative about the increased risk of device insertion failure.

Flap infection with dehiscence was the second most frequent cause of major complication, a little different from the literature, where this is the most frequent complication^7^, ^9^, ^10^, ^16^. Care in making the flap is important to minimize complications; we suggest some shapes which improve blood supply. In the anterior base flap, the blood supply comes from the superficial temporal artery and the dermal plexus; in the inferior base flap this blood supply comes from retroauricular and occipital branches of the external carotid artery^18^, ^19^, ^20^, ^21^.

Cholesteatoma is a rare and late complication which affected 1.2% of our patients with a mean time of onset 45 months after the procedure. By 2006, there were only 35 cases of cholesteatoma arising from the cochlear implant surgery reported in the literature, with an incidence varying from 5.4% in adults to 1.4% in children^22^, ^23^. The incidence in adult and children of our sample was around 1%. In the study from Bibas et al.^23^, the mean time of symptom onset suggestive of cholesteatoma was 42 months, very similar to what we found. Hence the importance of a clinical follow up in the long run in implanted patients, especially in cases of breaking the canal wall and injury to the tympanic annulus, although cholesteatomas also affect patients without intraoperative injuries. It is believed that the cholesteatoma is a consequence of bone resorption in a very thin wall with a later migration of keratinocytes^23^.

## CONCLUSION

Surgical complications were very low in occurrence, and most are successfully treated. These results confirm that the cochlear implant is a safe procedure and most of the surgical complications can be treated with conservative measures or mild interventions.
